# Report on Dental Pathology and Surgery

**Published:** 1870-12

**Authors:** W. H. Atkinson

**Affiliations:** Chairman


### REPORT ON DENTAL PATHOLOGY AND SURGERY

BY W. H. ATKINSON, CHAIRMAN.

### PATHOLOGY.

Pathology in the large sense includes all functional activity, whether of a healthy or diseased character. In the differential sense it has been restricted to the latter.

All apprehension of difference of health and disease is but the perception of the phases of production, destruction, and reconstruction of bodies and processes, in equation of presentment in health, and predominance of or tendency to overbear this equation, or balancing of processes in disease.

The constitution of a body, out of which arises the possibles in temperament and diathesis, must be understood to enable an observer to recognize harmonious and inharmonious play of plan and process in the functions of forming and maintaining in place the primates of which the body or process is composed.

Bodies are formed, fed, and worked upon a plan, by a process, for an end or purpose, whether they be planets, plants, or peoples, in mineral, vegetable, or animal modes of presentment.

Differentiation.—Without orderly arrangement of points of likeness and unlikeness in bodies and processes, we can have no intelligible standard of differences.

All the observations capable of being made without optical instruments are coarse and ambiguous; and hence the classification of bodies thus discernible is vague and unscientific, as the text-books of natural history abundantly prove.

Since the perfecting of the microscope, naturalists have broken camp on the old battle ground of germs and species, and transferred their opposing onslaughts to that of class and kingdom.

The characteristic of germs and species has ever been unmeaning and occult, but that of class and kingdom is even less conclusive and less clear.

The cataloguing of any character of sameness, likeness or unlikeness involves a process of perception, which is itself the product of the mechanism of sense, by which that which perceives elaborates the sentient process.

Essential or necessary conditions are posited at the avenues of sense as guards against error and misjudgment resulting upon the misunderstanding of any part or the harmony of the entire and complete process of perception of fact and philosophy.

Aberrant-action of any sense, or conjunction, or ellipsis of one or more senses, vitiates, obscures, aborts or prevents the mental conclusion. The complete conclusion of fact and philosophy, or the demonstration, as it has been called, can only occur by wedding the territories of matter and mind.

That this may clearly appear, it is only necessary to state —mental labors or events occur in *consciousness*, and physical or bodily events occur in *space*.

How shall we untie without cutting this gordian knot, of bringing together in consentaneous harmony these as yet parallel lines, infinitely projected and hence rigidly indifferent? The categories of primates in consciousness and physics may be thus annotated or tabulated, viz:

Consciousness has as basis or ground, mind, spontaneity, force.

Physics has as basis or ground, matter, space, effect.

The wedding of all these seeming indifferents in the territory of *time* completes the conditions of individual existence. *Existence*, then, being the mutual saturation of three positives and three negatives, namely: mind, spontaneity, force, and matter, space, effect, may be regarded in finite or infinite degrees.

Individual *ex*-istence as an infinite is incomprehensible; but as an infinitude of infinitesimals composing the universe in the great sense, is the only rational basis of realty of bodily presence.

All bodies must come into existence out of the unpronounced oceans of force and form, in accordance with the limitations of these in the type upon which they are built.

This involves necessity of production and maintenance, which simply means creation or procreation, growth and nutrition. This last word nutrition involves the idea of change in the constituents of the body or bodies that elaborate function. And just here is the point in which all the past has been more or less at fault in interpretation of the where, the when, the how of function.

Function is the performance of process, whether that be mental or material, and each must partake of the other to constitute it function at all. So it becomes apparent that all of science and philosophy depends upon the appearance and disappearance of oneness and diversity throughout the whole range of cognizable being and not being, or existence and non-existence of presence, or in other words the metamorphoses occurring in planets, minerals, or plants and animals, in the multifarious process of feeding and being fed, from world to bathybius the extremes of hardness and softness between which anthropology makes its advent to explain the whole.

In the main the differentiations that mark slightest dissimilarity of a diatom or some infinitesimal body has engaged the attention of naturalists and microscopists, thus tarrying in thinnest matters of detail, and spending whole lives in building up unimportant differences into impassable barriers to all but those who were able not only to follow but transcend these wonderful hair splitters.

Why should the asserted identity of appearance under the microscope of an air bubble and a globule of mercury be a stumbling block in the way of the veriest novice, only that superficial observers have laid down a superficial authority which dominates so many who are essaying to study microscopy from books?

The power to differentiate is evidently an endowment of mind, and the mind is the concurrent result of all means of perception; is the full play of all the senses in normal action.

The senses acquaint us with the qualities of things. That is to say, resistance is the primal necessity to the sense of feeling.

Solution is the prerequisite to taste, fineness of division or volatility to smell, vibration to hearing, and impact of light to sight, each retaining the essential condition of contact specialized in accordance with the differential necessities of each mechanism to metamorphose touch into idea, thought, opinion, belief, and knowledge by which we become informed upon the various subjects which engage our attention.

If, then, as is now apparent, we can only exercise complete powers of body and mind by being in possession of them, and also having a consciousness of that possession to enable us to exercise them in specific direction, how absolutely necessary is it that this exercise voluntary and spontaneous be untrammeled, regular, full and free in every department, and in the entire rounding out of demonstration of indisputable knowledge! Every effort at systemizing the greatest and minutest differences with which natural science has thus far been favored has fallen in the main far short of this clearness of demonstration, and hence we are and have been overloaded with mere detail and crudities, that will continue to obstruct true progress so long as these remain uncancelled and unarranged.

And now let us propound the basis upon which the future structure of natural science must be built to attain the clearness sought: 1st, crystal; 2d, cell; 3d, body; out of which the three kingdoms or domains naturally take their origin, and upon which they are firmly set. To understand the mineral dominion, *crystalosophy* must be the field of study. If we would comprehend the vegetable domain we must make a differential study of *cellosophy*. Both of these are necessary to introduce us into the domain of *corpusculosophy*, which defines the measure of endowment of the animal.

*Crystalosophy*, *cellosophy*, and *corpusculosophy* constitute the tripod upon which natural philosophy may now stand erect as their embodiment and assertion in the complete dominion of matter and mind as interdependent necessities of substance and force.

Investigators of natural phenomena may be divided into two distinct classes of observers, namely, the *ex*clusives and *in*clusives.

The *ex*clusives have held the field from the earliest times, and still hold it in all accepted teachings of the books and the schools.

The *in*clusives have been so few and obscure as to have been overslaughed or ignored in every age; the exclusives appropriating their labors and clipping them of their real significance and greatest use by differentially cataloguing them as their own.

The two great examples of exclusives holding control of the domain of natural philosophy are nominated physicists and vitalists, neither allowing the other the credit that each lays claim to, hence the irrepressible conflict of the war now waging between them, only to be settled by the *in*clusives taking the field and showing the necessity, utility and harmony of both aspects of this unity in diversity displayed in physical and vital phenomena.

"Observers" are limited by their *powers*, and "investigators" by their *means*.

If we would clearly understand this, we must be able to define and propound both "powers" and "means."

The ultimate power of observation is nothing less than consciousness, hence our consciousness will be clear and definite, or clouded and indefinite, in accordance with the completeness or incompleteness of our mental machinery.

In the strict sense, then, this machinery is a "means" of consciousness, but not the only means; for appliances and apparatus belonging to optics and mechanics are also essential "means" by which our "powers" are increased and made definite in extent and certainty.

Orderly arrangement of observations, then, may be said to constitute investigation when legitimately compared.

The order of impressions becoming completed, consciousness or knowledge must be known before we can by possibility differentiate legitimacy from its opposite.

This order will be more clearly apprehended by explaining a diagram representing the several stages of mental labor, from inception to culmination of the process, by which alone we can possibly know!

Whether mind precedes body in order of existence, or body mind, need not much perplex us, as we must study them together as we find them, to make any progress worth the effort.

We know that the body comes into existence by a serial order of gestational processes, upon a definite plan, and that the mind becomes educated by the inception and repetition of mental exercises.

The bodily machinery wears out and falls into decay, so the mental mechanism becomes incapable of recalling past experiences, which is called forgetting.

A mass of soft iron, while constituting a link in the magnetic circle, is a magnet of power proportionate to its quantity and the strength of the charging current; but the instant the chain is broken the iron is non-magnetic as before.

Did the iron or the current constitute it a magnet? Neither alone, but both in conjunction!

The mass of mind now extant, alas! is analagous to the soft iron, only capable of clear mental perception when in connection with some other mind whose exercise has given it the requisite strength to communicate the current.

Without the power of consciousness, no touch, taste, smell, sound or sight can be interpreted into sense or understanding. So it is plain that reason and unreason, sense and nonsense, are but the matchings and mismatchings of events in the territory of consciousness.

And herein lies the difficulty of propounding the results of observation and investigation in the territories of matter and mind.

Instead of acknowledging that they are interdependent, we have assumed that they are distinct domains that might persist independently.

We might as well affirm that respiration, circulation, and innervation could be separately maintained.

All organization is the result of alternate domination and submission, or compromise of dominion of two tendencies, namely: centripetality and centrifugality, or togetherness and apartness.

The full dominion of the former in planetary substance results in crystal; of the latter in ether. The full or complete dominion of togetherness in the territory of consciousness results in knowledge; that of apartness in diffuse feeling.

Rapidly passing from one of these states or extremes of tension to the other, without noting the intermediate stages of physical or mental progress, has given rise to the doctrine of cataclysms or floodings, in which all traces of stages of togetherness and apartness were obliterated, and a new order instituted in accordance with the whereabouts of the observers when they gave attention to the status in which they were.

This doctrine, without the shadow of support in nature, has withstood progress to such an extent that teachers to-day dare not depend upon their own convictions to verify the truth of the propositions they essay to investigate, but cringingly ask "what the authorities will think," or "in what book the proof may be found?" As if authority and book were anything but the repetition or the record of the mental labor necessary to the demonstration.

Tidal-waves, upheavals and explosions have been cited as proofs of cataclysm as the order of natural planetary progress; each and every one of which is substantial testimony to the opposite doctrine of regular, serial, order, in the occurrence of planetary processes.

In what, then, does our power to observe, investigate, and to differentiate consist? Manifestly, first, in possessing the power; and second, in exercising it in accordance, not only with the laws upon which it is itself constituted, but also in agreement with the laws and conditions of the subject of observation or investigation.

As the world progresses, ought we not, then, to be better and better prepared to solve, piece by piece, the problem before us? How shall we proceed to conserve helps and banish or surmount hurts to our progress?

In accord with that already stated, our first need is a correct mental philosophy to enable us to acquire the power of knowing when our queries are satisfactorily answered, so that we may rise above the adverse magnetism of those teachers who dwell with emphasis upon a single state or department of the labor of explaining the solution of the problem by varying the statement and in endless repetition, until we are engulfed in the mælstrom of voice without significance; till from very weariness we are forced to acknowledge that to be clear which is not explained, but rendered yet more intensely dark!

The veriest skepticism is preferable to such a state!

Mercury and carbonic acid are examples of bodies capable of passing from the vaporous to the crystaline state or condition by the management of pressure alone, so that crystal, soft-solid, waxy fluid, liquid and gaseous bodies may be exhibited of these substances running up and down the scale of togetherness and apartness without cataclysm, flood or lines of demarkation of these differentiations, which are thus found to exist but in degrees commensurate to the pressure put on or taken off at the pleasure of the investigator, or the spontaneity of natural production! *Pragmatic pathology*

includes all the progresses and regresses in the constructions and destructions of the individual (separate) domains of crystalosophy, cellosophy and corpusculosophy, an exhaustive observation of whose processes will bring within the pale of consciousness all the possibilities of matter and mind.

In a former report it was asserted that the human kingdom was built upon the destructions of the mineral, vegetable and animal kingdoms, by correlating them on a higher and finer order of arrangement in the human as their predestinate ultimatum.

In like manner the pragmatic pathology includes all the former phases of solidal, humoral (or fluidal), neural and cellular presentments of this predestinate (or completed, pronounced), pragmatic pathology; the study of which will reduce to scientific certitude all that was of authority in the former pathologies, and effectually reconcile the discrepancies that have kept the territories of consciousness and physics perpetually asunder.

That the blissful reign of the pragmatic pathology may soon prevail, it is necessary that it be understood and taught by as many as possible at the beginning of its pronouncement.

I account myself fortunate in the happy opportunity that our young, fresh and nearly untrammeled profession presents for the introduction of the latest, highest and best discoveries of law and process; no less than the triumphant vindication in practice of the new method of securing the reproduction of carious and necrosed bones repeatedly brought before this body in my former reports, and exhibited in detail to many who are present.

